# Impact of a Season of Bike Patrol on Police Officers’ Level of Fitness: A Pilot Study

**DOI:** 10.3390/ijerph18126214

**Published:** 2021-06-08

**Authors:** Frédérique Lehouillier, Marc-Olivier Dugas, Martin Lavallière

**Affiliations:** Program of Kinesiology, Department of Health Sciences, Université du Québec à Chicoutimi, Saguenay, QC G7H 2B1, Canada; frederique.lehouillier1@uqac.ca (F.L.); marc-olivier.dugas1@uqac.ca (M.-O.D.)

**Keywords:** bike patrol, work demand, cardiovascular test, fitness, fit for duty

## Abstract

Bike patrollers must have a good level of fitness to perform their patrolling duties adequately and effectively by bike and accomplish specific work tasks, which may require the use of various physical capacities. However, there is little information on the real workload associated with bike patrol and its impact on health. The purpose of this study was to assess the general physical fitness of police officers before and after a season of bike patrolling and then quantify its effects on each patroller’s health. All six male police officers (29.5 ± 4.3 years old) performed two complete physical fitness evaluations (PRE- and POST-season), which included anthropometric measurements (weight, waist circumference, and body mass index), a push-up test, a sit-up test, a grip strength test, a vertical jump test, a sit-and-reach test, and an aerobic capacity test on a bicycle ergometer. Paired *t*-tests were used to evaluate the differences in test performance between the PRE- and POST-season. Grip strength, estimated VO_2_max, and power deployed on the bike all showed significant improvement after the season (*p*-value 0.0133; 0.007; and 0.003, respectively). No significant differences were found among the evaluation’s other components (*p* > 0.05). Results show the workload associated with a bike patrol season caused a considerable improvement in grip strength, VO_2_max, and power deployed on the bike, and might be beneficial for their overall health as a work-integrated avenue to keep the officers fit for duty. Further research on the subject is suggested.

## 1. Introduction

To achieve their role of protecting citizens, bike patrollers must be in good physical condition to preserve their own health and safety as well as the health and safety of the communities they serve. Indeed, their cardiovascular and muscular capacity must be sufficiently developed to perform their patrolling duties adequately and effectively by bike and accomplish specific work tasks, which may require the use of strength or various physical capacities. Compared to automobile patrolling, bike patrolling requires a considerable physiological effort by increasing the physical workload required to perform similar tasks [1]. For instance, driving a vehicle has been documented as a light physical activity and close to most sedentary activities, whereas biking is estimated at a moderate to vigorous effort level [2]. Combining driving sedentary behavior with the fact that police officers’ physical fitness tends to decline with the number of years since graduation, an older officer who starts bike patrolling might be at a higher risk of developing musculoskeletal disorders (MSD) while practicing his or her profession [3]. Depending on the type of bike patrol, many variables—such as the position on the bike, the repetitive movement required in biking, and the intensity of the patrols—could cause physical discomfort or even injure an officer who is unfit over time [4,5,6,7]. In addition, since some patrollers may not have the cardiovascular or muscular capacity to maintain the effort required for bike patrols, the execution of specific work tasks will require a higher aerobic demand for these individuals [8]. This can result in significant physiological fatigue, which could reduce the physical performance of the officer over time [8,9,10]. However, such a patrol is an opportunity to have police patrollers more physically active while on duty, and, therefore, reduce the burden on their health associated with the sedentary activities of car patrol.

Bike patrol is typically used to conduct community policing and, by doing so, brings the police departments closer to citizens and their community. The use of a bike while patrolling makes police officers less intimidating to citizens by removing the patrol vehicle, which can be seen as a physical barrier, and promotes interaction between citizens and police officers [11]. With the use of a bike while patrolling, police officers can cover events, festivals, and any area inaccessible by car more effectively, such as parks and bikeways. Its general usability is similar to foot patrol but enables one to generate faster speeds and cover longer distances in general or for a specific destination. In larger cities, bike patrol is also used for traffic control with the goal of either blocking a street or managing traffic [5]. In a dense urban environment, this means of transportation becomes an advantage since it is not as restrictive as many other vehicles, having less road conspicuity and avoiding certain road signs, thus the officer can arrive at an intervention faster [5]. Since they become their own means of transportation, bike patrollers must be in good physical condition to take advantage of all the benefits surrounding bike use. Despite proper equipment and training, patrollers may be unable to fulfill their duties due to a lack of strength, muscle power, or an insufficient cardiovascular capacity. Moreover, bike patrollers all have different tasks, equipment, and work practices depending on their jurisdictions since they are not carried out for the same purpose or in the same geographical context [12]. Despite the lack of knowledge and scientific publications on this profession, it is known that organizational practices differ with regard to the minimum requirements needed to become a police officer or to maintain such employment over time [13]. The typical series of physical tests used by police organizations mainly consists of the same tests, but the minimum requirements for these tests differ greatly from one institution to another [13]. For that reason, officers from different regions who technically perform relatively similar duties can present a wide array of physical conditions. Myers et al. [14] demonstrated that police officers from two different American police organizations had different physical fitness regardless of their age. This irregularity in fitness requirements raises questions as to what the real physical requirements to be a police officer are, particularly so in specific units such as bike patrols.

For instance, in Canada, there is no documented physical evaluation for hiring bike patrollers. To address this issue, the Police Advisory Board of England and Wales (PABEW) assessed a total of 20 bike patrollers (17 males and 3 females) already in service to establish new minimum physical requirements for employment [15]. In a 2010 report, the PABEW recommended increasing the minimum requirements on the MultiStage Fitness (a shuttle test) to level 5 (completed) for all bike patrol officers. This test result corresponds to an estimated VO_2_max of 36.1 mL/kg × min^−1^ and is categorized as a very poor to poor VO_2_max for men ages 20 to 39 [15,16]. The VO_2_max has been defined as the highest amount of oxygen used by the body during strenuous exercise [17]. As this profession requires a significant cardiovascular capacity, patrollers should have more than acceptable aerobic fitness [1]. Moreover, the PABEW did not fully describe the profession when establishing the physical and physiological demands and thus issued requirements according to the actual demand for the profession that might not be sufficient. These evaluations were also carried out in a single cohort of patrollers and do not allow the observation of whether the sample fairly represents the bike patroller population.

Takken et al. [1] studied the physiological demands of the bike patrol profession. A total of 16 male and 4 female officers from the Utrecht police department (Netherlands) wore sternal heart rate sensors and a Polar watch for three 8-h work shifts to calculate the number of TRIMP (training impulses) developed during the bike patrols. The TRIMP unit is a unit of measurement that quantifies exercise workload according to a percentage of maximum heart rate and exercise duration [1]. They measured a higher score of TRIMP in officers carrying out bike patrols (2429 TRIMP units/week) than among professional cyclists (2000 TRIMP units/week). This shows that the workload needed for bike patrols exceeded the physiological stress threshold measured in professional cyclists in addition to having average VO_2_peak measurements well below those professionals [1]. With such physical and physiological demands, police officers carrying out bike patrol should have an adequate level of aerobic fitness to reduce the risk of fatigue that could prevent them from doing their job safely and adequately [10].

Without knowing the specific work tasks of the job and its associated demands, it is difficult, and maybe even impossible, to know how much effort is required, therefore, making it more difficult to intervene in health and safety with these officers. Because of the numerous benefits of bike patrols mentioned above, the use of bike patrol squads is increasing in numerous police organizations [5]. Despite the knowledge of the many benefits of regular physical activity in health [18], an unfit officer might react poorly to the intensities required to sustain the volume of physical activity in bike patrolling [1,16]. Moreover, there is little to no information on the real workload associated with this job and its impact on the patrollers’ health [1]. Therefore, the purpose of this study was to assess the general physical fitness in police officers before and after a summer of bike patrol in order to quantify its effects on the patroller’s health (PRE- vs. POST-season). The main hypothesis was that the physical fitness of the bike patrollers would improve over the course of the 12-week bike patrol season.

## 2. Materials and Methods

### 2.1. Participants

A total of 6 male police officers (*n* = 6) between the ages of 26 and 38 years old (mean = 29.5 ± 4.3 years old) from a Quebec (Canada) police department participated in this study. The sample represented the entirety of the bike patrol cohort of the Police Organization. No women took part in the study because none were employed as bike patrollers by the police department at the time of the data collection. All participants provided written consent and a Physical Activity Readiness Questionnaire (PAR-Q) before enrolling in this study [19]. Approval for the study’s procedures was obtained from the Ethical Research Committee (Ref.: 2020–262) before the start of the study. All the patrollers were paid for their participation since the study took place during their work shifts, which were hours covered by their employer.

### 2.2. Measures

A total of two full physical fitness evaluations were conducted for each participant: one before (PRE) and one after (POST) the bike patrol season, which began in early June and ended in late August (for a total of 12 weeks in function). The evaluations included anthropometric measurements (body weight, waist circumference, and body mass index (BMI)), tests assessing muscular strength, endurance and power (1 min push-ups, 1 min sit-ups, grip strength, and vertical jump) in addition to a flexibility test (sit-and-reach) and an aerobic capacity test on a bicycle ergometer (ramp test). These evaluations were all carried out by 2 certified kinesiologists in the police station’s training facilities. In addition to these evaluations, a questionnaire on recreational physical activity during the last 7 days was distributed to the officer’s PRE- and POST-season to determine whether the police officer carried out other physical activities apart from bike patrol. An individual description of every evaluation completed is described below.

#### 2.2.1. Anthropometric Measurements (Weight, Waist Circumference and Body Mass Index)

Bodyweight was measured with a scale (SECA 700). Waist circumference was taken with a flexible tape placed at the right iliac crest before positioning the rest of the tape horizontally around the officer’s waist, passing over the left iliac crest and returning to the starting point [16]. When the tape was properly positioned around the waist, a measure (to the nearest 0.1 cm) was taken after 2 normal expirations. BMI was calculated based on self-reported height and cross-checked on the driver’s license and weight measurement [16].

#### 2.2.2. Push-Ups

A maximum push-up test specific to law enforcement modified to fit a duration of 1 min was used to assess upper-body muscular endurance [20,21,22]. The starting position consisted of having arms fully extended, hands placed on the ground slightly wider than the shoulders with the fingers pointed forward, and legs together (high position). The participant was instructed to keep his body in a straight line during the entire duration of the test. When ready, the 1 min timer began, and the officer lowered his body by bending his elbows until his chest touched a 9.5 cm high object placed directly under his chest to then extend his elbows and return to a high position. The completion of the movement counted as 1 repetition, and the officer completed as many repetitions as possible in the 1-min time frame.

#### 2.2.3. Sit-Ups

A maximal 1-min sit-up test was used to assess abdominal muscular endurance [13,21,22,23]. The starting position consisted of maintaining a supine position, knees bent at 90°, and feet flat on the ground. The participant’s hands were placed behind his neck with his fingers crossed, while 1 evaluator kept the participant’s feet on the ground during the test. When ready, the 1 min timer began, and the officer flexed his trunk, lifting his shoulders off the floor until his elbows touched his knees to then extend his trunk to go back to his starting position. This chain of movements counted as one repetition. The officer completed as many repetitions as possible in the 1 min time frame while keeping the correct positioning.

#### 2.2.4. Grip Strength

Overall upper-body strength was evaluated with a grip strength test [16,24]. The dynamometer (Jamar Smedley Hand Dynamometer) was adjusted thus that the 2nd metacarpal of the 3rd finger (middle finger) was properly placed in the center of the handle. The participant was asked to stand with his elbow in complete extension (180°) close to his thigh without touching it, to keep his forearm in a neutral position, and to start squeezing while exhaling. When ready, the participant squeezed the handle with his hand as hard as he could for 5 s and then released. Two trials were completed for both hands. The result was the sum of the best measurement for each hand.

#### 2.2.5. Sit-and-Reach

Lower back and hamstring flexibility were assessed with a sit-and-reach test [13,16]. The test involved flexing the trunk as much as possible while using a sit-and-reach box to measure flexion distance. The officer had to remove his shoes before sitting on the floor with his feet against the box, knees extended, and feet hip-width apart. The location of the participant’s feet was at 23 cm in the testing box. The participant was instructed to keep his knees and arms extended, his hands pronated and overlapped, all while flexing the trunk gently forward and pushing the slide with his fingertips and holding the position for 2 s. The trial was not counted when the participant’s knees flexed and/or when the movement was jerky. The best measurement of the 2 trials was kept for analysis.

#### 2.2.6. Vertical Jump

Vertical jump was used to assess lower-body muscular power and was calculated with the *My jump 2* application [25]. Prior to the test, measurements of the length of the leg from the greater trochanter of the femur to the tip of the foot (with the ankle in full extension) and the height of the hips in the optimal position for a vertical jump (distance between the hip and the floor when the legs are bent at 90°) were taken. From the starting position with feet at shoulder width apart and hands on hips, the participant was asked to jump as high as possible while keeping his feet pointed horizontally and knees and hips extended. The participant’s hands had to remain on his hips throughout the movement. The iPad’s camera was used to record the jump. After setting the start and end of the jump on the recording interface, the officer’s measurements were entered in the application to calculate the height of the jump. The best of 2 trials was considered for analysis. The lower body power (power developed during the jump) was then calculated using the Sayers equation as shown below [26]:

Peak Power (watts) = 60.7 × jump height (cm) + 45.3 × body mass (kg) − 2055(1)

#### 2.2.7. Ramp Test

A ramp aerobic capacity test was performed to determine the participant’s power developed on the bike and the maximal physiological effort that can be deployed by the officers. The entire procedure of the test was explained, and heart rate and blood pressure were measured manually before and after the test. The ramp protocol began with a 5-min warm-up at 100 watts [27,28,29]. The bike’s workload was then increased by 15 watts every minute until the officer reached maximum effort and stopped or until the officer could not support the workload (watts) demanded by the test. When the participant reached his maximum effort, the test stopped, and the workload was reset to 100 watts in order to perform a 4-min cooldown. No rotations per minute (rpm) were imposed for the test, but the officer had to maintain the workload (bearing power in watts) required for the test. The workload was modulated with the help of the PERFPRO Studio training software paired with a TACX Vortex smart trainer. The bike used was a single speed LAGER brand to avoid any snagging of speed brackets during the test. A sternal heart rate sensor was used throughout the test to measure the maximal heart rate (RS800, POLAR). The maximal power deployed on the bike during the tests was represented using a watt/kg ratio, which is a ratio of the maximal aerobic power (MAP) and weight of the officer [30]. With these data, the estimated VO_2_max was calculated using the American College of Sports Medicine (ACSM) formula as shown below [26]:

VO_2_max (ml/min/kg) = (10.8 × Power (watts))/Body mass (kg) + 7 (2)

#### 2.2.8. Physical Activity Questionnaire

A modified version of the Godin–Shephard Leisure-Time Physical Activity Questionnaire (GSLTPAQ) was distributed to the officers PRE- and POST-season [31]. The modified version was used to seek information on the number of hours per week instead of the number of times per week spent doing mild, moderate, and strenuous physical activity outside of work. This self-administered questionnaire was applied to determine whether the police officer carried out physical activities other than bike patrol during the season and how much time was spent doing those activities.

### 2.3. Statistical Analysis

Mean and standard deviation was used to describe the participants’ physical fitness and performance outcomes on every test. The normality of each variable’s distribution was evaluated with a Shapiro–Wilk test, and all data were distributed normally. The F-Test Two-Sample was then used to test for homogeneity of variance, which was proven homogenic (equal) for every test. A paired *t*-test was performed to observe the differences in test performance between the start and end of the bike patrol season (PRE- vs. POST-season). The effect size was calculated to measure the strength of the outcome relationship between the start and end of the patrol season (week 1 vs. week 12). Modified Hedges’ g formula for effect size was used due to a small sample size (*n* = 6). The significance level was defined as *p* < 0.05 for every test. All statistical analyses were conducted using SPSS for Windows (version 24.0, IBM, US).

## 3. Results

The results are shown in Table 1. Bodyweight, waist circumference, and BMI showed no significant difference (*p*-value 0.1658; 0.4015; 0.1448, respectively). Grip strength, estimated VO_2_max, and maximum power deployed on the bike all showed significant improvement after the season (*p*-value 0.0133; 0.007; and 0.003, respectively). However, grip strength showed a small effect size of g = −0.2749, while estimated VO_2_max and power deployed on the bike showed a large effect size of g = −1.0391 and g = −1.2902, respectively. These effect size values are negative since the POST-season’s measurements were higher than the PRE-season’s, which shows an improvement in the officer’s physical fitness after the patrol season. No significant differences were found between PRE- and POST-patrol seasons for the other components of the evaluation (*p* >0.05).

The results regarding the Leisure-Time Physical Activity Questionnaire [31] are presented in Table 2. On average, police officers increased the number of hours spent performing leisure-time physical activity at the end of the season when compared to before the season. However, there was no correlation between the recreational physical activity improvement observed in POST-season and the officers’ VO_2_max values difference between POST- and PRE-season, with a value for R^2^ = 0.0992.

## 4. Discussion

The purpose of this study was to assess the variation in the fitness of bike patrollers over a 12-week period in order to quantify the impact of the patrol on their physical conditions. Our findings showed that bike patrollers significantly improved their estimated VO_2_max, power deployed on the bike, and grip strength, while push-ups, sit-ups, sit-and-reach, vertical jump, and lower-body power decreased over the 12 weeks of patrolling. Despite slight improvements, weight, waist circumference, and BMI did not improve significantly.

Although the anthropometric values (mass, waist circumference, and BMI) were not significant, these measurements were still lower at the end of the bike patrol season. Therefore, they might have had several benefits for the general health of police officers. The average police officer’s BMI, based on the ACSM classification of disease risk, went from “overweight” to “normal” after 12 weeks of patrol [16]. A lower BMI poses less risk to their overall health [32]. In addition, the average decrease in waist circumference of more than 1 cm indicates a visceral adiposity reduction and a better distribution of fat mass, which is another indication of good health [16]. It is widely recognized that police officers present important cardiovascular risks [18]; these results, even if not significant, might suggest that bike patrollers could improve their body composition in the course of their work and thus reduce their risk of developing cardiovascular diseases in the future [33]. Knowing that the prevalence of obesity among a sample of Quebec police officers (2099 males and 756 females) varied between 21.1% for men and 7.3% for women and that their overall health was categorized at moderate risk of cardiovascular disease, even a slight improvement of these health factors can be beneficial for police officers [18]. It is noteworthy that the patrol season was only 12 weeks and that a longer period of work could produce higher benefits for these components.

Among our sample, the sit-up test mean was 28.3 repetitions (POST), which places patrollers at the 30th percentile of police officers based on previous work (Table 1) [13]. Despite being ranked as moderate, one could argue this result represents a low level of abdominal muscle endurance, considering the importance of this muscle group for many police work tasks, including interventions that require the use of force. As part of a specialized profession such as bike patrollers, and since abdominal muscle health is strongly linked to back health (more specifically the lumbar region), core endurance should be developed to reduce the risk of injury during the long periods of physical effort required by this employment [1,34]. Crawley et al. observed a ceiling effect on the push-up and sit-up performances after the second half of a fitness program (week 8 to 16) and, therefore, suggested that more attention should be directed to this period to continue increasing the overall effectiveness of cadet physical training [35]. In this current study, a ceiling effect can also be observed for these same tests, possibly due to a lack of muscular training requested as well as a time restriction of these tests, which limits the number of repetitions that can be performed. The high standard deviation of the push-ups for both the PRE- and POST-season shows a great variation in the fitness level among the police officers in this cohort. Despite this variation between officers, over the course of the patrol season that lasted 12 weeks, the bike patrol did not seem to influence core endurance and upper-body strength.

Although men are known to be less flexible, several police work tasks require flexibility, such as bike dismounting and all movements requiring bending and reaching [36]. In this study, the sit-and-reach mean result (Table 1) places the patrollers between the 1st and 10th percentile of police officers [13]. This low level of flexibility could reveal stiffness of the hamstrings and glutes, which can increase the intervertebral pressure of the lower back and ultimately lead to low back pain [37]. With tight muscles in the posterior part of the leg, the pedaling biomechanics could alter the proper positioning of the policemen on their bikes and prevent them from maintaining an anterior rotation of the pelvis while cycling, which is only possible with the appropriate activation of the glutes and hamstrings. In addition, the normal biomechanics of the pedaling movement will also change with the appearance of low back pain by reducing the range of motion in multiple articulations [38]. If the pedaling movement cannot be performed properly while seated on the bike due to a lack of flexibility, or if mobility is restrained due to the officer’s duty belt and its associated tools and equipment, the police officer could become at a much greater risk of developing musculoskeletal disorders. Introducing flexibility exercises before and during the patrol season would be beneficial to help police officers reduce the muscle tension caused by the high level of physical activity from bike patrolling. Considering that the lower back region is known to be weaker among the police population, it would be interesting to measure the strength and endurance of the lumbar region in specialized police officers, such as bike patrollers, to help determine whether the physical demand for the job presents health risks for the officers [39]. The riding position throughout the season could also be evaluated in future studies to assess whether the particular positioning of the officers on their bike exposes them to a higher prevalence of low back pain or other discomfort [40,41].

According to the ACSM, a VO_2_max of 41.0 mL/kg × min^−1^ is considered to be acceptable for 20- to 39-year-old men. Bike patrol requires a significant cardiovascular workload, thus the VO_2_max of these police officers could be higher to allow them a better physiological maneuver margin to fulfill their job safely and efficiently [1]. The estimated VO_2_max before the patrol season was 40.0 mL/kg × min^−1^, which is below the acceptable value established by the ACSM for this age group. Since wearing a bullet-proof vest increases the physiological demand of effort associated with work, a low VO_2_max could exacerbate the workload demand placed on them and accelerate fatigue and physical exertion, leading to higher risks of developing injuries/or pain due to muscle fatigue [10,42,43]. In fact, Knapik et al. showed that low-fit military recruits who completed a preconditioning program before their basic combat training tended to have lower injury risk compared to recruits of similar fitness who did not complete such program [44]. The development and implementation of physical preparation programs for the bike patrol seasons would help alleviate these health risks for patrollers by improving cardiovascular and muscular capacity prior to engaging in such specialized units.

During the season, the mean estimated VO_2_max increased by more than 8 mL/kg × min^−1^, and the power developed on the bike went from 3.3 watts/kg to 4.2 watts/kg (Table 1). During their numerous bicycle patrols, the officers increased their volume of physical activity associated with duty rapidly, which led to a gain in cardiovascular fitness and muscle power in the lower limbs. Prior to being assigned to the bike patrol, many of the patrollers self-reported having not pedaled on a bike for a few years nor having engaged in regular physical activity. It is possible that these police officers obtained greater improvement in their cardiovascular and muscular capacity than the more trained patrollers [45]. However, despite seeing an increase in the number of hours spent doing leisure physical activity at the end of the season when compared to before the season, the current results showed no correlation between this increased time in POST-season and the officers’ VO_2_max values difference between PRE- and POST-season. Therefore, more work needs to be conducted in order to precisely identify which portion of the gains are associated with the bike patrollers’ related physical activity, their leisure physical activity [31], or a synergetic effect of both.

A police officer’s cycling experience can play an important role in performance since past experience might influence how they manage their energy while cycling to an intervention by adapting their position and cycling techniques depending on the context [1,3,5,46,47]. By having more hours spent on a bike, an officer’s pedaling movement should become more fluid, which could increase biomechanical efficiency and help reduce the associated physiological demand [30]. With these gains, a police officer’s level of fatigue during typical tasks could be reduced, which would decrease the likelihood of using excessive force in interventions, posing less disability risk to the officer and its organization and, most importantly, prevent heart diseases and many health problems [13,16]. Nevertheless, these improvements in a bike patroller’s physical condition do not mean that a pre-season physical preparation can be omitted. On the contrary, it proves that the fitness requirements for bike patrol are underestimated and need to be taken more seriously. Bike patrollers must have good acquisition of cycling techniques and be in good physical condition, in the same way, that vehicle patrollers are not undertaking their first hours of driving when they are hired [5]. Practical and technical training should, if not already in place, be implemented to enable current and future bike patrollers to practice bicycle techniques [5] and reduce the health risks [18].

### Limitations

This study was carried out with a small sample (*n* = 6) of all-male bike patrollers. The police organization where this research took place only employs a total of six bike patrollers to patrol their territories, which made us unable to increase the number of participants. Beyond this limitation, there were no women assigned to bike patrol during data collection. Considering that women may have a lower physical condition when compared to men, it would be interesting to observe the impacts of a bike patrol season on their physical capacities [46]. In addition, a comparison with the entire police department is not currently possible and, therefore, does not allow us to know whether the current sample composed of the bike patrol cohort is representative of the police officers working in this organization.

In addition, this study occurred in the summer of 2020 during the COVID-19 pandemic. Without access to the various university research laboratories due to their closure for sanitary measures, the research team had to use indirect measures to assess VO_2_max. Future projects should prioritize direct measures of gas consumption and cycling performance in the laboratory to ascertain the current results. Moreover, the police organization in question reported a kilometer (km) reduction covered by the bike patrollers compared to previous years because of the COVID-19 pandemic. Indeed, for the period during which the current research took place, a total of 1660 km were covered by the 6 officers in the three months of bike patrolling (June 2020 to August 2020). From 2017 to 2019, 4794, 2281, and 3025 km were traveled by the patrol for the same number of police officers and time periods, respectively. With these major differences in distance traveled per period, the work itself and its demand on officers were necessarily different and suggested a lower physical and physiological demand. Moreover, the current data did not control the pedaling intensity or time spent on bicycles of each participant, as the author felt this could have affected the outcome of the study. All of these restrictions could have greatly influenced the results of this study and should be taken into consideration.

## 5. Conclusions

As an entry-point to allowing better contact with the population [11], bike patrolling is an important situational asset for police institutions, allowing better contact with the public and contributing to a good physical condition among police officers. In this present work, bike patrollers significantly increased their cardiovascular health and lower body strength in the course of their duty. Moreover, the low level of aerobic capacity (PRE), low back flexibility (PRE and POST), and abdominal muscle endurance (PRE and POST) observed in bike patrollers poses a health and safety issue since bike patrolling requires a much greater cardiovascular capacity and puts them at risk of developing musculoskeletal disorders [1]. In the future, bike patrols could help police organizations address health-risk problems present among police officers who expose themselves to a higher risk of cardiovascular disease and premature death [18,32,33]. Hence, not only should appropriate conditioning programs be put in place to improve physical ability before the patrol season and reduce physical risks, but this type of patrol should also be seen as an opportunity to develop a “new police” in their community. More studies on bike patrolling are suggested to help understand the physiological demand of this profession and to evaluate its impact on the health and safety of police officers to intervene better and prevent hazardous work conditions when feasible.

## Figures and Tables

**Table 1:** Comparison of the officers’ physical condition between the PRE- and POST-bike patrol season.

Measures	PRE (*n* = 6)		POST (*n* = 6)		T Value (D.O.F. = 5)	*p*-Value	Effect Size
	Mean	ST.D.	Mean	ST.D.			
Body Weight (kg)	83.6	13.4	82.2	12.4	1.622	0.1658	0.0697
Height (m)	1.82	0.10	1.82	0.10	-	1.0000	-
Waist Circumference (cm)	90.8	8.0	89.5	6.2	0.916	0.4015	0.1132
Body Mass Index (m/kg^2^)	25.2	2.0	24.8	1.6	1.729	0.1448	0.1529
Push-ups (repetition)	36.5	21.3	35.5	17.0	0.519	0.6268	0.0341
Sit-ups (repetition)	28.7	7.5	28.3	8.3	0.154	0.8840	0.0276
Grip strength (kg)	104.2	19.1	111.9	17.6	−3.749	0.0133 ^a^	−0.2749 ^b^
Sit-and-reach (cm)	28.3	14.2	27.6	14.1	1.136	0.3075	0.0308
Vertical Jump (cm)	38.4	5.8	37.9	5.6	0.478	0.6538	0.0585
Lower Body Power (watts)	4012.2	750.8	3965.1	682.5	0.572	0.5921	0.0429
Estimated VO_2_max (ml/kg × min^−1^)	40.0	5.6	48.7	5.4	−7.420	0.0007 ^a^	−1.0391 ^c^
Power (watt/kg)	3.3	0.5	4.2	0.4	−6.607	0.0003 ^a^	−1.2902 ^c^

ST.D.: Standard deviation, D.O.F.: Degree of freedom. ^a^ Indicates a significant difference between week 1 and week 12 of patrol season (*p*-value < 0.05). ^b^ Indicates a small effect size. ^c^ Indicates a large effect size.

**Table 2:** Comparison of the number of hours spent on recreational physical activity between the PRE- and POST-bike patrol season.

Participants	PRE Total Hours (*n* = 6)	POST Total Hours (*n* = 6)	Variation (Post—Pre) (Hours)
1	2	7	5
2	4	14	10
3	2.5	2.5	0
4	4	7.5	3.5
5	1	3	2
6	5.5	12	6.5
Mean	3.17	7.67	4.5
ST.D.	1.63	4.64	3.52

ST.D.: Standard deviation.

## Data Availability

The data presented in this study are available on request from the corresponding author.
